# Missing Skeletal Muscle Metastases of Papillary Thyroid Carcinoma

**DOI:** 10.3390/diagnostics10070457

**Published:** 2020-07-06

**Authors:** Leszek Herbowski

**Affiliations:** Neurosurgery and Neurotraumatology Department, District Hospital, 71-455 Szczecin, Poland; leszekherbowski@data.pl

With regard to several important gaps in the work “Skeletal Muscle Metastasis in Papillary Thyroid Microcarcinoma Evaluated by F18-FDG PET/CT” [1], it seems necessary to clarify and extend some key issues in this text.

First of all, one should agree with authors of this paper that “… one must bear in mind the fact that muscle metastasis from PTC is possible …”. Indeed, based on epidemiological data, I have estimated the occurrence of papillary or follicular thyroid carcinoma metastasis to skeletal muscles at a few cases per billion people. Thus, the probability of such exceptional metastases approaches zero, but this phenomenon does happen.

Secondly, the authors cited several cases of papillary thyroid carcinoma metastases to skeletal muscles including one more case added by themselves. The total number of cases of dissemination of PTC to skeletal muscles reported by the authors equals 13, based on the analysis of medical literature from the years 2006–2016. Certainly there are more such cases because not all diagnosed cases of rare diseases are willingly published. Nevertheless, having analyzed medical literature as far back as the last hundred and ten years, I have identified in total 58 cases of papillary (PTC) and follicular (FTC) thyroid cancer metastases to skeletal muscles: 45 cases in the considered time range 2006 through 2016, and 13 cases from 1907 to 2005 (doi: 10.3892/ol.2018.8216). Still, considering only the cases of papillary thyroid cancers (PTC) during this period (2006–2016), a total of 34 skeletal muscle metastases were described in 27 patients: 12 females (44%) and 15 males (56%) [2,3,4,5,6,7,8,9,10,11,12,13,14,15,16,17,18,19,20,21,22,23,24,25,26,27]. The mean age was 54.7 years (95% CI: 47.6–61.8; range: 25–89). There were no significant age differences between women and men (*t*-test: *p* = 0.96).

Thirdly, based on the 34 cases scrutinized in my paper, the most frequent PTC metastatic muscles were the erector spinae, gluteus and sternocleidomastoid muscle (Table 1). Although the sternocleidomastoid muscle seems to be frequently prone to PTC metastasis, after excluding three jatrogenic cases of carcinoma seeding only one natural sternocleidomastoid muscle metastasis is left [24].

Fourthly, in a period of over one hundred years, only three cases of skeletal muscle metastases from papillary thyroid microcarcinoma were reported [17,23,28]. The first case involved the pterygoid, masseter and temporal muscles [17], the second the deltoid muscle [23] and the third the psoas muscle [28]. Hitu et al. added the next case of microcarcinoma of the papillary thyroid spreading to the gluteal muscle which was diagnosed by F18-FDG PET/CT study.

The rarer the occurrence of a given medical phenomenon, the harder and more reliable work analyzing the past should be done by the authors. In the light of the facts demonstrated here, it is difficult to consider the theses presented in the paper by Hitu et al. as reliable and consistent with prior research. The accuracy of the previously described medical cases’ analysis is based not only on the review of the medical database in terms of various categories, but above all on finding an appropriate algorithm that allows us to understand the information present but not necessarily directly specified in the text. That is why it is so important to supervise the team collecting and analyzing previously published works. Hence, the uniqueness of casuistic study is not to show that it is an exceptional work, only to prove that similar previous research consists of single cases; and I can confidently put forward a thesis that the entire review process has failed in this specific situation. Therefore, to improve the quality and validity of published work, it is necessary to supervise the construction of the research at subsequent stages of its creation and reviewing.

## Figures and Tables

**Table 1:** Localization of 34 skeletal muscle metastases of papillary thyroid carcinoma (PTC).

No	Muscles	PTC Metastases
1	Erector spinae	4
2	Gluteus muscles	4
3	Sternocleidomastoid	4
4	Pterygoid	3
5	Trapezoid	2
6	Masseter	2
7	Thigh muscles	1
8	Piriformis	1
9	Pelvic	1
10	Deltoid	1
11	Psoas/Iliopsoas	1
12	Rectus abdominis	1
13	Biceps	1
14	Vastus	1
15	Adductor longus	1
16	Biceps femoris	1
17	Paraspinal	1
18	Temporal	1
19	Subscapularis	1
20	Gastrocnemius	1
21	Latissimus dorsi	1
	**Total**	**34**
